# Matrilysin (MMP-7) as a significant determinant of malignant potential of early invasive colorectal carcinomas

**DOI:** 10.1054/bjoc.2001.1790

**Published:** 2001-05

**Authors:** T Masaki, H Matsuoka, M Sugiyama, N Abe, A Goto, A Sakamoto, Y Atomi

**Affiliations:** First Department of Surgery and Department of Pathology, Kyorin University, 6-20-2, Shinkawa, Mitaka City, Tokyo 181-8611, Japan

**Keywords:** matrilysin, colorectal carcinoma, budding, tumour invasion, metastasis

## Abstract

Matrix metalloproteinases play a crucial role in tumour invasion and mestasis. Matrilysin (MMP-7) has been shown to correlate with nodal or distant metastasis in colorectal carcinomas; however, its implication in early invasive colorectal carcinomas has not been determined. This study was undertaken to clarify the association of matrilysin expression with clinicopathologic parameters in early invasive colorectal carcinomas. 38 early invasive colorectal carcinomas treated by local excision or radical surgery were examined. Tumour budding was evaluated as the number of dedifferentiation units along the entire invasive margin. Matrilysin protein levels were determined using immunohistochemical study. Univariate analysis showed that matrilysin expression alone was significantly associated with distant metastasis (*P*= 0.0339), and both tumour budding and matrilysin expression were significantly associated with adverse outcome (*P*= 0.0005, 0.0341). Histological differentiation, vessel invasion, and depth of invasion were not significantly associated with either distant metastasis or adverse outcome. Multivariate analysis confirmed that tumour budding and matrilysin expression were independently associated with adverse outcome, although the significance of matrilysin expression was marginal (*P*= 0.0488). Tumour budding at the invasive margin and matrilysin expression are more useful in identifying high-risk groups for adverse outcome in patients with early invasive colorectal carcinomas. © 2001 Cancer Research Campaign www.bjcancer.com


British Journal of Cancer (2001) **84**(10), 1317–1321
© 2001 Cancer Research Campaign
doi: 10.1054/ bjoc.2000.1790, available online at http://www.idealibrary.com on IDEAL®

### Matrilysin (MMP-7) as a significant determinant of malignant potential of early invasive colorectal carcinomas

**T Masaki[1], H Matsuoka[1], M Sugiyama[1], N Abe[1], A Goto[2], A Sakamoto[2] and Y Atomi[1]**

First Department of Surgery[1] and Department of Pathology[2], Kyorin University, 6-20-2, Shinkawa, Mitaka City, Tokyo 181-8611, Japan

**Summary** Matrix metalloproteinases play a crucial role in tumour invasion and mestasis. Matrilysin (MMP-7) has been shown to correlate with nodal or distant metastasis in colorectal carcinomas; however, its implication in early invasive colorectal carcinomas has not been determined. This study was undertaken to clarify the association of matrilysin expression with clinicopathologic parameters in early invasive colorectal carcinomas. 38 early invasive colorectal carcinomas treated by local excision or radical surgery were examined. Tumour budding was evaluated as the number of dedifferentiation units along the entire invasive margin. Matrilysin protein levels were determined using immunohistochemical study. Univariate analysis showed that matrilysin expression alone was significantly associated with distant metastasis ($P = 0.0339$), and both tumour budding and matrilysin expression were significantly associated with adverse outcome ($P = 0.0005, 0.0341$). Histological differentiation, vessel invasion, and depth of invasion were not significantly associated with either distant metastasis or adverse outcome. Multivariate analysis confirmed that tumour budding and matrilysin expression were independently associated with adverse outcome, although the significance of matrilysin expression was marginal ($P = 0.0488$). Tumour budding at the invasive margin and matrilysin expression are more useful in identifying high-risk groups for adverse outcome in patients with early invasive colorectal carcinomas. © 2001 Cancer Research Campaign http://www.bjcancer.com

**Keywords**: matrilysin; colorectal carcinoma; budding; tumour invasion; metastasis

For the last two decades, endoscopic polypectomy has been widely used for the treatment of neoplastic lesions of the large intestine, and this technique is now assumed to be satisfactory for the treatment of colorectal adenomas. However, it is still controversial whether endoscopic polypectomy is suitable once carcinomatous cells penetrate the muscularis mucosae (Colacchio et al, 1981; Cooper, 1983; Morson et al, 1984; Haggitt et al, 1985; Cranley et al, 1986; Christie, 1988; Muto et al, 1991). Lymph node metastasis, residual carcinoma, local recurrence and hepatic involvement have been reported to occur in about 6%, 6%, 4% and 1% of cases of early invasive colorectal carcinomas removed by polypectomy (Furusawa, 1985; Muto et al, 1991). To reduce the adverse outcome of these cases, many researchers have advocated the following histological criteria; massive submucosal invasion, vascular invasion, or poorly differentiated histology, when considering additional surgery after endoscopic resection of early invasive colorectal carcinomas (Morson et al, 1977; Colacchio et al, 1981; Haggitt et al, 1985; Furusawa, 1985; Muto et al, 1991). However, the positive predictive value of these histological features is rather low, about 20%, and a more useful risk factor is needed in the clinical setting.

The proteolytic degradation of extracellular matrix by matrix metalloproteinases (MMP) has been shown to be one of the essential events in tumour invasion and metastasis (Stetler-Stevenson, 1993; Crawford and Matrisian, 1995). Matrilysin (MMP-7) is a member of the MMP gene family, and has proteolytic activity against a wide spectrum of substrates such as collagens, proteoglycans, elastin, laminin, fibronectin and casein (Woessner and Taplin, 1988; Miyazaki et al, 1990; Wilson and Matrisian, 1996). It is produced by malignant tumour cells such as oesophageal, gastric, colorectal, head and neck, lung, prostate and hepatocellular carcinomas (Yamamoto et al, 1997; Adachi et al, 1998; Yamashita et al, 1998; Adachi et al, 1999; Babletz et al, 1999; Yamamoto et al, 1999). Immunohistochemical studies have shown that the expression of matrilysin correlates significantly with nodal or distant metastasis in gastric and colorectal carcinomas (Adachi et al, 1998–1999; Yamashita et al, 1998). In oesophageal squamous cell carcinomas, matrilysin expression is associated with the depth of tumour invasion, advanced tumour stage, recurrence, and recurrence within the first postoperative year; however, no significant association is seen between matrilysin expression and nodal or distant metastasis (Yamamoto et al, 1999). These studies were based on univariate analysis; therefore, no definite conclusion could be obtained about the implication of matrilysin expression with reference to clinicopathologic parameters. Furthermore, no previous studies were performed focusing on early invasive colorectal carcinomas. This study was undertaken to clarify the association of matrilysin expression with clinicopathologic characteristics of early invasive colorectal carcinomas using univariate and multivariate analysis.

*Received 28 July 2000*
*Revised 15 January 2001*
*Accepted 25 January 2001*

*Correspondence to:* T Masaki

### PATIENTS AND METHODS

### Patients

38 patients with early invasive colorectal carcinoma treated by local excision or radical surgery at the First Department of

Surgery, Kyorin University between 1994 and 1999 were examined retrospectively. 11 patients underwent local excision initially (endoscopic mucosal resection, 5; transanal resection, 4; transsacral resection, 2), and additional bowel resection with lymph node dissection was attempted in 6 patients after histological examination of the resected specimens. The remaining 27 patients underwent radical surgery (bowel resection with lymph node dissection) initially. 27 were men and 11 were women. The mean age was 66 years with a range of 40 to 85 years. The depth of invasion was submucosal in all tumours. The tumour location was the rectum in 14, the left colon in 12, and the right colon in 12 patients. Patients' clinical records and pathological reports were reviewed with special attention to the presence or absence of lymph node metastases, local recurrence and distant metastases.

### Tissues

From each resected specimen, one archival formalin-fixed paraffin-embedded tissue block containing normal mucosa was selected for immunohistochemical study. 5-μm-thick paraffin sections were cut from each block, and mounted on lysin-coated slides.

### Immunohistochemistry

Immunostaining for matrilysin was performed using a standard avidin-biotin peroxidase technique. Sections were deparaffinized in xylene, and hydrolysed with 100%, 90%, 80% and 70% ethanol. The antibody used was anti-hMMP-7 mouse monoclonal antibody (clone 141-7B2; Fuji Chemical, Toyama, Japan). This antibody is specific for human pro-MMP-7, and does not cross-react with human active-MMP-7, MMP-1, -2, -3, -8, -9 or -13. As the antigen retrieval procedure for matrilysin, the sections were heated to 121˚C in an autoclave for 15 min. Endogenous peroxidase was blocked with 0.3% $H_2O_2$ in PBS (30 min), and the sections were rinsed in PBS. Non-specific staining was eliminated by 30 min of incubation with normal horse serum. The sections were incubated at 4˚C overnight with primary antibody (1:50, 10 $\mu g\ ml^{-1}$), at 37˚C with biotinylated secondary antibodies for 30 min, and with avidin-biotin complex for 30 min. Peroxidase reaction was visualized with a solution of DAB (diaminobenzidine tetrahydrochloride). The sections were counterstained with haematoxylin, dehydrated and mounted.

### Evaluation of matrilysin expression

Immunostaining signals at the invasive margin were counted, and the scores were calculated as the number of stained cells divided by the total number of carcinoma cells at the invasive margin. Cases were considered positive when >30% of carcinoma cells at the invasive margin were stained with the antibody, according to a previous report (Yamamoto et al, 1999).

### Evaluation of clinicopathological parameters

A consecutive section from each specimen was stained with haematoxylin and eosin. Tumours were evaluated for predominant grade, lymphatic vessel invasion, blood vessel invasion, regional lymph node involvement, surgical margin status, presence or absence of adenomatous component, and presence or absence of dedifferentiated histology at the invasive margin, the finding of a single cancer cell or a solitary trabecular form along the entire invasive margin, which was identical to 'budding' (Hase et al, 1993) (Figure 1). For objective evaluation, the number of dedifferentiation units along the entire invasive margin was counted under light microscopy, and graded into 4 categories; none (0 unit), mild (1~20 units), moderate (21~50 units) and severe (≧ 51 units) according to a previous publication (Ono et al, 1996). The depth of submucosal invasion was graded into 3 levels, as described previously (Masaki and Muto, 2000).

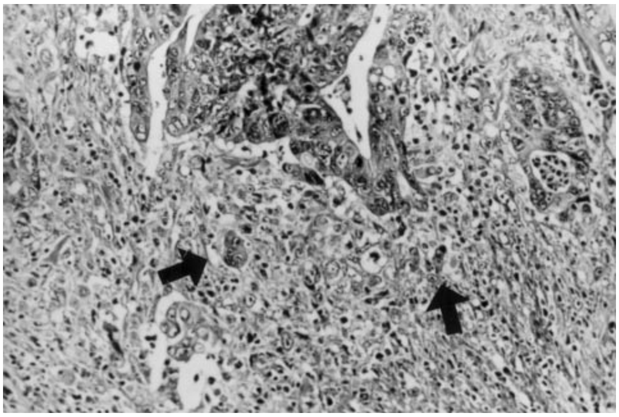

**Figure 1** Representative photograph of tumour budding at the invasive margin. Small nests of cancer cells with poorly differentiated histology at the invasive margin of the tumour (arrows). H&E, ×50

### Statistical analysis

The associations between matrilysin expression and clinicopathologic parameters were examined using $\chi^2$ (chi-squared) test or Fisher's exact test. The associations between adverse outcome and clinicopathologic parameters including matrilysin expression were analysed using multiple logistic regression analysis (software, SPSS for Macintosh, version 6.1 (Chicago, IL, USA)). A *P* value less than 0.05 was accepted as statistically significant.

### RESULTS

### Demographic features of 7 patients with adverse outcome

As shown in Table 1, lymph node metastases were found in the radically resected specimens in 3 patients, and distant metastases in 3 patients. One patient had local recurrence, although he had undergone 'potentially curative' trans-anal resection for a 20-mm rectal tumour one year before. This recurrence was assumed to be due to residual lymphatic vessel involvement.

### Matrilysin expression

Figure 2 shows representative results of matrilysin staining in early invasive colorectal carcinomas. The cytoplasm and cell membrane of carcinoma cells were stained for matrilysin, but stromal cells other than some monocytes or surrounding normal mucosa were not stained. In this study, matrilysin expression was positive in 13 of 38 tumours (34%) examined (Table 2).

 © 2001 Cancer Research Campaign

**Table 1** Demographic details of 7 patients with adverse outcome

| Sex | Age | Location | Size (mm) | Shape | Treatment | Histology | sm–inv | ly | v | adenoma | LN met | Local rec | Distant met |
|---|---|---|---|---|---|---|---|---|---|---|---|---|---|
| M | 47 | Rectum | 20 | S | LE | W | 1 | – | – | – | – | + | – |
| M | 75 | Rt. Colon | 8 | P | LE+RS | M | 2 | + | – | – | + | – | – |
| M | 71 | Rectum | 17 | U | RS | P | 3 | – | – | + | + | – | – |
| F | 74 | Lt. Colon | 40 | P | RS | W | 1 | – | – | + | + | – | – |
| M | 73 | Rectum | 30 | P | RS | W | 1 | – | – | – | – | – | Liver (8 m) |
| M | 61 | Rectum | 15 | S | LE | W | 3 | – | + | – | – | – | Bone, lung (2 y) |
| M | 73 | Rt. Colon | 12 | S | RS | W | 3 | – | – | – | – | – | Lung (4 y) |

M, male; F, female; P, pedunculated; S, sessile; U, ulcerated; LE, local excision; RS, radical surgery; W, well differentiated; P, poorly differentiated; sm-inv, level of submucosal invasion; ly, lymphatic invasion; v, venous invasion; adenoma, presence/absence of adenomatous component; LN, lymph node; met, metastasis; rec, recurrence.

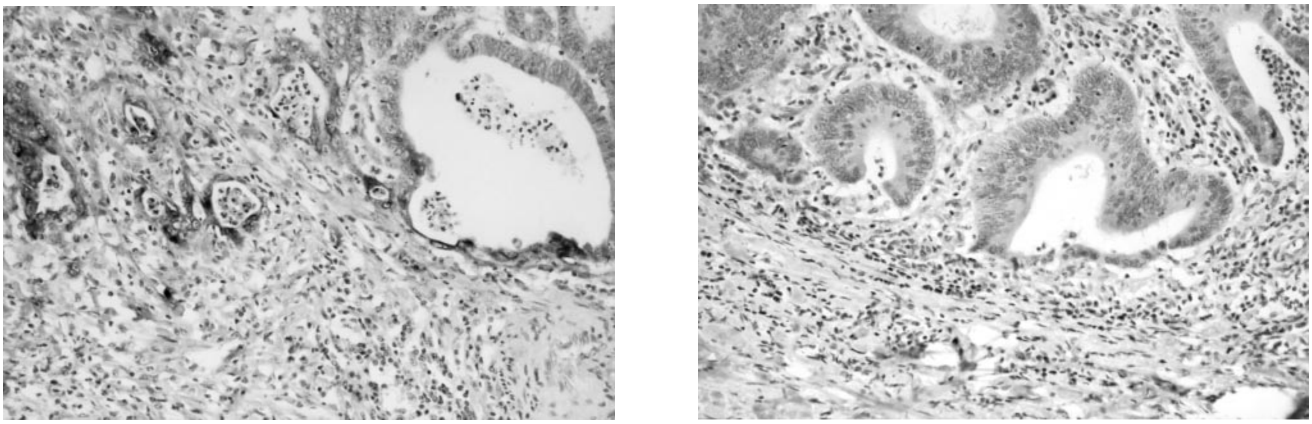

**Figure 2** Representative photographs of matrilysin expression in early invasive colorectal carcinomas. (**A**) Matrilysin is strongly positive at the invasive margin, ×50. (**B**) Matrilysin is negative in a rectal submucosal carcinoma, ×50

**Table 2** Association of matrilysin expression with clinicopathologic parameters

| | | Matrilysin expression | | |
|---|---|---|---|---|
| Variables | Category | Positive | Negative | *P* |
| Histological differentiation | Well | 12 | 20 | |
| | Moderate | 1 | 3 | NS |
| | Poor | 0 | 2 | |
| Ly | Negative | 12 | 21 | |
| | Positive | 1 | 4 | NS |
| V | Negative | 11 | 19 | |
| | Positive | 2 | 6 | NS |
| Depth of invasion | sm1 | 4 | 8 | |
| | sm2 | 1 | 4 | NS |
| | sm3 | 8 | 13 | |
| Tumour budding | None | 2 | 10 | |
| | Mild | 9 | 8 | |
| | Moderate | 1 | 5 | NS |
| | Severe | 1 | 2 | |
| Distant metastasis | Negative | 10 | 25 | |
| | Positive | 3 | 0 | 0.0339 |
| Adverse outcome | Negative | 8 | 23 | |
| | Positive | 5 | 2 | 0.0341 |

NS, not significant; Ly, lymphatic invasion; V, venous invasion.

*© 2001 Cancer Research Campaign*

**Table 3** Patients' outcome and clinicopathologic parameters

| | | Distant metastasis | | | Adverse outcome | | |
|---|---|---|---|---|---|---|---|
| Variables | Category | Present | Absent | *P* | Present | Absent | *P* |
| Histological differentiation | Well | 3 | 29 | | 5 | 27 | |
| | Moderate | 0 | 4 | NS | 1 | 3 | NS |
| | Poor | 0 | 2 | | 1 | 1 | |
| Ly | Negative | 3 | 30 | | 6 | 27 | |
| | Positive | 0 | 5 | NS | 1 | 4 | NS |
| V | Negative | 2 | 28 | | 6 | 24 | |
| | Positive | 1 | 7 | NS | 1 | 7 | NS |
| Depth of invasion | sm1 | 1 | 11 | | 3 | 9 | |
| | sm2 | 0 | 5 | NS | 1 | 4 | NS |
| | sm3 | 2 | 19 | | 3 | 18 | |
| Tumour budding | None | 0 | 12 | | 0 | 12 | |
| | Mild | 2 | 15 | | 4 | 13 | |
| | Moderate | 0 | 6 | NS | 0 | 6 | 0.0005 |
| | Severe | 1 | 2 | | 3 | 0 | |
| Adenomatous component | Negative | 0 | 8 | | 2 | 6 | |
| | Positive | 3 | 27 | NS | 5 | 25 | NS |
| Matrilysin expression | Negative | 0 | 25 | | 2 | 23 | |
| | Positive | 3 | 10 | 0.0339 | 5 | 8 | 0.0341 |

NS, not significant; Ly, lymphatic invasion; V, venous invasion. Adverse outcome includes lymph node metastasis, local recurrence and distant metastasis.

**Table 4** Multivariate analysis

| Variable | Odds ratio | *P* value |
|---|---|---|
| Tumour budding | 13.8 | 0.0338 |
| Matrilysin expression | 51.6 | 0.0488 |

Model chi-squared 16.013, df 2, $P = 0.0003$.

### Matrilysin expression and clinicopathologic parameters

As shown in Table 2, matrilysin expression was positive in 5 of 7 tumours (71%) with an adverse outcome, and in 8 of 31 tumours (26%) without an adverse outcome. This difference was statistically significant ($P$ = 0.0341, Fisher's exact test). Matrilysin expression was positive in all 3 tumours (100%) with distant metastasis, and in 10 of 35 tumours (29%) without distant metastasis. This difference was also statistically significant ($P$ = 0.0339, Fisher's exact test). Matrilysin expression was not significantly associated with histological differentiation, vessel invasion, depth of invasion and grade of tumour budding.

### Adverse outcome and clinicopathologic parameters

Univariate analysis showed that matrilysin expression alone was significantly associated with distant metastasis ($P$ = 0.0339, Fisher's exact test), and both tumour budding and matrilysin expression were significantly associated with adverse outcome ($P$= 0.0005, 0.0341; Table 3). Histological differentiation, vessel invasion, and depth of invasion were not significantly associated with either distant metastasis or adverse outcome. Multivariate logistic regression analysis using tumour budding and matrilysin expression as independent variables confirmed that both factors were independently associated with adverse outcome, although the significance of matrilysin expression was marginal (Table 4).

### DISCUSSION

Tumour budding is a histological finding that tumour cells are dissociating from the main tumour. Hase et al and Ono et al have recently reported that the presence of microscopic clusters of undifferentiated cancer cells at the invasive front of the lesion is one of the most useful factors for identifying groups at high risk of haematogeneous or regional metastasis among patients with advanced colorectal cancer (Hase et al, 1993; Ono et al, 1996). In an earlier study, we demonstrated that tumour budding was more useful in predicting lymph node metastasis in early invasive colorectal carcinomas than the following histological criteria; massive submucosal invasion, vascular invasion or poorly differentiated histology (Masaki and Muto, 2000). In this study, we showed that tumour budding was significantly associated with adverse outcome, i.e. lymph node metastasis, local recurrence or distant metastasis in early invasive colorectal carcinomas, suggesting that tumour budding could represent malignant potential of early invasive colorectal carcinomas in a broader sense. In this study, tumour budding was evaluated objectively by counting the number of focally dedifferentiated cancer cell nests or a single cancer cell at the invasive margin, and graded into none, mild, moderate and severe according to Ono et al's publication (Ono et al, 1996). Although the association of tumour budding with adverse outcome was statistically significant in this study, the frequency of adverse outcome was not completely parallel with the grade of tumour budding. As shown in Table 3, none of 6 tumours (0%) with moderate budding had an adverse outcome; however, 4 of 17 tumours (23%) with mild budding had an adverse outcome, suggesting that the number of focally dedifferentiated cancer cell nests or a single cancer cell alone does not satisfactorily imply tumour aggressiveness.

Matrilysin (MMP-7) is a member of the matrix metalloproteinases (MMP) and has a proteolytic activity against a wide spectrum of extracellular matrix (Woessner et al, 1988; Miyazaki et al, 1990;

 © 2001 Cancer Research Campaign

Wilson et al, 1996). Previous immunohistochemical studies have shown that matrilysin expression correlates significantly with nodal or distant metastasis in gastric and colorectal carcinomas (Adachi et al, 1998, 1999; Yamashita et al, 1998). However, these studies mainly examined advanced carcinomas, and the association of matrilysin expression with clinicopathologic parameters was studied using univariate analysis alone. In this multivariate logistic regression analysis, matrilysin expression was significantly and independently associated with distant metastasis or adverse outcome in early invasive colorectal carcinomas, confirming that matrilysin expression plays a crucial role in tumour invasion and metastasis at its earliest stage.

These immunohistochemical results can be applicable in clinical practice. Radical surgery with lymph node dissection or adjuvant chemotherapy should be given in about 10–15% of patients with early invasive colorectal carcinomas removed by polypectomy (Furusawa, 1985; Muto et al, 1991). Although histological criteria such as massive submucosal invasion, vascular invasion, or poorly differentiated histology have been widely accepted as risk factors in considering additional radical surgery (Morson et al, 1977; Colacchio et al, 1981; Furusawa, 1985; Haggitt et al, 1985; Muto et al, 1991), the positive predictive value of these factors was rather low, about 20%, and more useful risk factors have been looked for. The present study and our previous study (Masaki et al, 2000) suggested that tumour budding at the invasive margin and matrilysin expression are more useful in identifying high-risk groups for adverse outcome in patients with early invasive colorectal carcinomas.

A large prospective study is mandatory to confirm the usefulness of tumour budding and matrilysin expression in the management of early invasive colorectal carcinomas.

### ACKNOLWEDGEMENTS

We thank Drs Y Shimada, K Nunomura and S Uchiyama for providing tissue blocks. For expert technical assistance we thank Ms N Sato and K Ooshima.

### REFERENCES

Adachi Y, Itoh F, Yamamoto H, Matsuno K, Arimura Y, Kusano M, Endo T. Hinoda Y, Oohara M, Hosokawa M and Imai K (1998) Matrix metalloproteinase matrilysin (MMP-7) participates in the progression of human gastric and esophageal cancers. *Int J Oncol* **13**: 1031–1035

Adachi Y, Yamamoto H, Itoh F, Hinoda Y, Okada Y and Imai K (1999) Contribution of matrilysin (MMP-7) to the metastatic pathway of human colorectal cancers. *Gut* **45**: 252–258

Babletz T, Jung A, Dag S, Hlubek F and Kirchner T (1999) β-catenin regulates the expression of the matrix metalloproteinase-7 in human colorectal cancer. *Am J Pathol* **155**: 1033–1038

Christie JP (1988) Polypectomy or colectomy? Management of 106 consecutively encountered colorectal polyps. *Am Surg* **54**: 93–99

Colacchio TA, Forde KA and Scantlebury VP (1981) Endoscopic polypectomy: inadequate treatment for invasive colorectal carcinoma. *Ann Surg* **194**: 704–707

Cooper HS (1983) Surgical pathology of endoscopically removed malignant polyps of the colon and rectum. *Am J Surg Pathol* **7**: 613–623

Cranley JP, Petras RE, Carey WD, Paradis K and Sivak MV (1986) When is endoscopic polypectomy adequate therapy for colonic polyps containing invasive carcinoma? *Gastroenterology* **91**: 419–427

Crawford HC and Matrisian LM (1995) Tumor and stromal expression of matrix metalloproteinases and their role in tumor progression. *Invasion Metastasis* **14**: 234–245

Furusawa M (1985) Prognosis of patients with colon cancer limited to submucosal layer, who had undergone endoscopic polypectomy. *Stomach Intestine* **20**: 1087–1094, (in Japanese with English abstract)

Haggitt RC, Glotzbach RE, Soffer EE and Wruble LD (1985) Prognostic factors in colorectal carcinomas arising in adenomas: implications for lesions removed by endoscopic polypectomy. *Gastroenterology* **89**: 328–336

Hase K, Shatney C, Johnson D, Trollope M and Vierra M (1993) Prognostic value of tumor “budding” in patients with colorectal cancer. *Dis Colon Rectum* **36**: 627–635

Masaki T and Muto T (2000) Predictive value of histology at the invasive margin in the prognosis of early invasive colorectal carcinoma. *J Gastroenterol* **35**: 195–200

Miyazaki K, Hattori Y, Umenishi F, Yasumitsu H and Umeda M (1990) Purification and characterization of extracellular matrix-degrading metalloproteinases, matrin (Pump-1), secreted from human rectal carcinoma cell line. *Cancer Res* **50**: 7758–7764

Morson BC, Bussey HJR and Samoorian S (1977) Policy of local excision for early cancer of the colorectum. *Gut* **18**: 1045–1050

Morson BC, Whiteway JE, Jones EA, Macrae FA and Williams CB (1984) Histopathology and prognosis of malignant colorectal polyps treated by endoscopic polypectomy. *Gut* **25**: 437–444

Muto T, Sawada T and Sugihara K (1991) Treatment of carcinoma in adenomas. *World J Surg* **15**: 35–40

Ono M, Sakamoto M, Ino Y, Moriya Y, Sugihara K, Muto T and Hirohashi S (1996) Cancer cell morphology at the invasive front and expression of cell adhesion-related carbohydrate in the primary lesion of patients with colorectal carcinoma with liver metastasis. *Cancer* **78**: 1179–1186

Stetler-Stevenson WAG, Aznavoorian S and Liotta LA (1993) Tumor cell interactions with the extracellular matrix during invasion and metastasis. *Ann Rev Cell Biol* **9**: 541–573

Wilson CL and Matrisian LM (1996) Matrilysin: an epithelial matrix metalloproteinase with potentially novel functions. *Int J Biochem Cell Biol* **28**: 123–136

Woessner JF Jr and Taplin C (1988) Purification and properties of a small latent matrix metalloproteinase of the rat uterus. *J Biol Chem* **263**: 16918–16925

Yamamoto H, Itoh F, Adachi Y, Sakamoto H, Adachi M, Hinoda Y and Imai K (1997) Relation of enhanced secretion of active matrix metalloproteinases with tumour spread in human hepatocellular carcinoma. *Gastroenterology* **112**: 1271–1277

Yamamoto H, Adachi Y, Itoh F, Iku S, Matsuno K, Kusano M, Arimura Y, Endo T, Hinoda Y, Hosokawa M and Imai K (1999) Association of Matrilysin expression with recurrence and poor prognosis in human esophageal squamous cell carcinoma. *Cancer Res* **59**: 3313–3316

Yamashita K, Azumano I, Mai M and Okada Y (1998) Expression and tissue localization of matrix metalloproteinase 7 (matrilysin) in human gastric carcinomas. Implications for vessel invasion and metastasis. *Int J Cancer* **79**: 187–194

© 2001 Cancer Research Campaign 
